# Genome-wide expression profiling establishes novel modulatory roles of vitamin C in THP-1 human monocytic cell line

**DOI:** 10.1186/s12864-017-3635-4

**Published:** 2017-03-23

**Authors:** Sakshi Dhingra Batra, Malobi Nandi, Kriti Sikri, Jaya Sivaswami Tyagi

**Affiliations:** Department of Biotechnology, All India Institute of Medical Sciences, New Delhi, 110029 India

**Keywords:** Vitamin C, THP-1, Macrophages, Microarray, Gene expression

## Abstract

**Background:**

Vitamin C (vit C) is an essential dietary nutrient, which is a potent antioxidant, a free radical scavenger and functions as a cofactor in many enzymatic reactions. Vit C is also considered to enhance the immune effector function of macrophages, which are regarded to be the first line of defence in response to any pathogen. The THP-1 cell line is widely used for studying macrophage functions and for analyzing host cell-pathogen interactions.

**Results:**

We performed a genome-wide temporal gene expression and functional enrichment analysis of THP-1 cells treated with 100 μM of vit C, a physiologically relevant concentration of the vitamin. Modulatory effects of vitamin C on THP-1 cells were revealed by differential expression of genes starting from 8 h onwards. The number of differentially expressed genes peaked at the earliest time-point *i.e.* 8 h followed by temporal decline till 96 h. Further, functional enrichment analysis based on statistically stringent criteria revealed a gamut of functional responses, namely, ‘Regulation of gene expression’, ‘Signal transduction’, ‘Cell cycle’, ‘Immune system process’, ‘cAMP metabolic process’, ‘Cholesterol transport’ and ‘Ion homeostasis’. A comparative analysis of vit C-mediated modulation of gene expression data in THP-1cells and human skin fibroblasts disclosed an overlap in certain functional processes such as ‘Regulation of transcription’, ‘Cell cycle’ and ‘Extracellular matrix organization’, and THP-1 specific responses, namely, ‘Regulation of gene expression’ and ‘Ion homeostasis’. It was noteworthy that vit C modulated the ‘Immune system’ process throughout the time-course.

**Conclusions:**

This study reveals the genome-wide effects of physiological levels of vit C on THP-1 gene expression. The multitude of effects impacted by vit C in macrophages highlights its role in maintaining homeostasis of several cellular functions. This study provides a rational basis for the use of the Vitamin C- THP-1 cell model, to study biochemical and cellular responses to stresses, including infection with *M. tuberculosis* and other intracellular pathogens.

**Electronic supplementary material:**

The online version of this article (doi:10.1186/s12864-017-3635-4) contains supplementary material, which is available to authorized users.

## Background

Human leukocytes and cultured cells of leukocyte origin can accumulate vitamin C (vit C) to millimolar concentrations, which is significantly above that in circulating blood where it is estimated to be in the range of about 50-100 μM, and of this at least 95% is in the reduced form [1–4]. Vit C is accumulated in mammalian cells by two types of transporters, namely, sodium-ascorbate co-transporters (SVCTs) for active transport of the reduced form and hexose transporters (GLUTs) for taking up the oxidized form, dehydroascorbate (DHA) [5]. Activated THP-1 cells rapidly accumulate vit C to millimolar concentrations, similar to activated RAW264.7 murine macrophages [6, 7]. Ascorbate is a cytosolic antioxidant and free radical scavenger that operates in concert with lipid-soluble membrane antioxidants, such asα-tocopherol or carotene, and may increase the ability of cells to cope with reactive oxygen metabolites generated by their activated phagocytic apparatus [8, 9]. Vit C also functions as a cofactor for enzymes involved in the biosynthesis of collagen [10, 11] and norepinephrine [12], and in the amidation of hormones [13]. Several studies have described that adequate circulating levels of vit C are consistent with a decreased risk of varied disease pathologies, such as stroke [14] or cardiovascular disease [15]. In this context, vit C supplementation has been reported to provide symptomatic relief and to enhance the expression of specific immune response markers [16]. It is noteworthy that most of the studies addressing the effects of vit C at optimum levels (70 μmol/l) on human health considered its supplementation together with other nutrients (usually zinc or within a multivitamin–multimineral formula), whilst a real understanding of its mechanism of action would possibly require its supplementation as a single component [17].

Mycobacteria-macrophage interactions have been characterized in primary as well as in vitro-differentiated cells to mimic the events that are considered to occur in vivo, namely, the entry and intracellular residence of *Mycobacterium tuberculosis* (Mtb) within alveolar macrophages [18–20]. Macrophage-like cell lines of human origin are considered as good models for in vitro-differentiated monocyte-derived macrophages [21]; moreover, they have the advantages of no donor variability of macrophage function, large numbers of cells can be grown reproducibly, cells can be studied at different stages (resting versus activated) and the cells closely model alveolar macrophages for processing of intracellular pathogens, for example Mtb-induced apoptosis [22]. Importantly, the human acute monocytic leukemia cell line, THP-1, develops macrophage functions following the addition of stimulators such as Phorbol myristate acetate (PMA) [23]. These differentiated THP-1 cells showed remarkable phenotypic changes *e.g.*, increased phagocytic activity and HLA-DR expression, increased complement receptor, Fc**γ**RI and Fc**γ**RII expression [24], CD11b, and CD14 [24, 25], indicating their similarity but not identity with mature human macrophages. THP-1 cells were found to be a suitable ex vivo infection model for studying Mtb-host interactions and anti-mycobacterials’ action on intracellular bacteria and yielded results comparable to that obtained using monocyte-derived macrophages (MDMs) [21].

We have reported earlier that tubercle bacilli treated with vit C develop an isoniazid-tolerant phenotype that is considered to be an indicator of bacterial dormancy. The vit C-induced drug tolerant response occurred in vitro as well as in infected THP-1 cells [26]. In view of the ability of vit C to induce a ‘dormant’ phenotype in Mtb, a temporal transcriptome profiling study of baseline gene expression to assess the response of THP-1 cells to vit C was undertaken. It was expected that such an analysis would pave the way for utilizing the vit C-based THP-1 infection model to study interactions of host cells with ‘dormant’ Mtb.

## Methods

### Cell culture conditions

THP-1 cells were grown to confluency in complete RPMI 1640 (Sigma-Aldrich^®^) medium (c-RPMI) supplemented with 2 mM glutamine, HEPES and sodium bicarbonate with 10% fetal bovine serum (HyClone™) and 1× Penicillin-Streptomycin solution (Sigma-Aldrich^®^) in 5% CO_2_, at 37 °C. Cell viability was checked by Trypan Blue exclusion and 99% viable cultures were used for gene expression studies.

### Gene expression analysis

Approximately, 8 × 10^6^ THP-1 cells were seeded per T-75 cm^2^ flask/20 ml c-RPMI in triplicate. Following differentiation with 30 nM phorbol myristate acetate (PMA) for 16–18 h, fresh c-RPMI was added and the cells were rested for 2–3 h. Subsequently, vit C (100 μM, Sigma-Aldrich^®^) was added to the treated flasks and not in the untreated (UT, control) flasks. At specified time-points (8, 24, 48 and 96 h) cells were harvested (vit C-treated and control flasks), the cell pellet was re-suspended in 1 ml TRI reagent (Molecular Research Center, USA) and stored at -80 °C for the isolation of RNA.

### RNA isolation and microarray analysis

Briefly, 1/10th volume of bromochloropropane (Molecular Research Center, USA) was added to thawed THP-1 lysates, vigorously shaken for 10 s and incubated for 10 min at room temperature. The samples were centrifuged at 12,000 rpm for 15 min at 4 °C. Precipitation was carried out in the presence of 1/100th volume of polyacryl carrier and isopropanol (0.6 volumes, for 30 min), centrifuged (12,000 rpm, 4 °C for 15 min), washed with 75% ethanol and then air-dried. Total RNA was dissolved in 100 μl of DEPC-treated water. Ten μg of total RNA was subjected to microarray analysis.

**Fig. 1:**
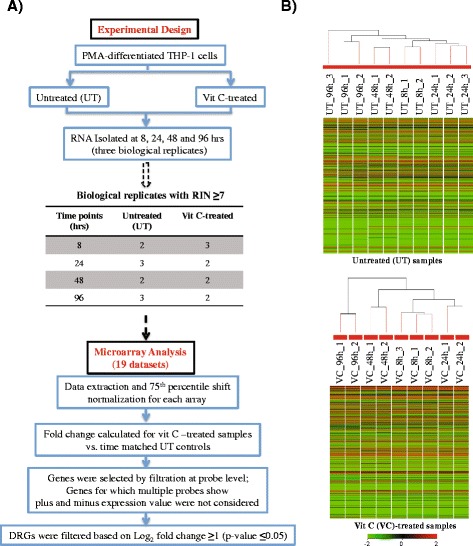
Microarray experimental and analysis details. **a** Flow plan for Experimental design and Microarray analysis, RIN indicates RNA Integrity number **b** Hierarchical clustering of log_2_ transformed 75th percentile normalized values for untreated (UT) samples and vit C-treated samples (VC) are shown, indicating the replicate confidence

A total of 24 RNA samples (3 biological replicates per condition) were analyzed for RNA integrity and 19 samples, whose RIN (RNA integrity number) was ≥7, were processed for microarray analysis (Fig. 1a). The samples were labeled using Agilent Quick Amp Kit. Briefly, 500 ng of total RNA was reverse transcribed using oligodT primer tagged to T7 promoter sequence. cDNA thus obtained was converted to double stranded cDNA and to cRNA in the in-vitro transcription step using T7 RNA polymerase enzyme. Cy3 dye was added into the reaction mix. cRNA was cleaned up using Qiagen RNeasy columns. Samples that passed the QC for specific activity were taken for hybridization. Labeled cRNA (600 ng) was hybridized to Human Whole Genome 8 × 60 K Array (Agilent Technologies AMADID: 027114) using the Agilent Gene Expression Hybridization kit at 65 °C for 16 h. The hybridized slides were washed and scanned on G2505C scanner. Data extraction from Images was done using Feature Extraction software Version 10.7 of Agilent Technologies.

### Microarray data processing and analysis

A plan of the microarray analysis is shown in Fig. 1a. Raw signal data from 19 expression arrays were log base 2-transformed and processed by 75th percentile shift normalization using the GeneSpring GX software (Agilent Technologies) as described [27–29]. Further, the robustness of data among the biological replicates for both untreated (UT) and vit C-treated samples was analyzed by hierarchical clustering (Fig. 1b). Next, expression fold change with *p*-values (student t-test) of all vit C treated conditions was calculated with respect to their time-matched untreated (UT) controls. Genes for analysis were selected by filtration at probe level. If there were multiple probes for the same gene, and they showed similar fold expression values (*i.e.* either all positives or all negatives), then the gene was selected for analysis, and where different probes for the same gene showed opposite fold expression values (positive and negative), that gene was removed from the analysis. Thus, at every time-point, ~5000 genes were not considered and ~ 25,000 genes were considered for analysis. Significant expression values were determined based on log_2_ fold change ≥ 1 with Benjamini-Hochberg FDR correction (q ≤0.05). Microarray experiments were performed at Genotypic Technology (Bengaluru, India) and the results are deposited at http://www.ncbi.nlm.nih.gov/geo/query/acc.cgi?acc=GSE73421. The biological significance of the gene expression modulation on vit C-treatment was analyzed using an online enrichment tool GOrilla (**G**ene **O**ntology en**RI**chment ana**L**ysis and visua**L**iz**A**tion Tool; http://cbl-gorilla.cs.technion.ac.il, [30]) using the input option of a single ranked list of genes on the basis of expression value. Gene descriptions explained in the results were obtained from Gene Cards® Human Gene database (http://www.genecards.org/cgi-bin/carddisp.pl?gene).

### Intracellular vit C estimation

For vit C estimation, DNPH method was used [31]. The experimental set up was the same as that for gene expression analysis and performed in triplicate. Briefly, at the individual timepoints (8, 24 and 48 h), THP-1 cells (UT and Vit C-treated) were scraped and harvested at 1200 rpm for 10 min. The pellet from two flasks was resuspended in 200 μl water and the suspension was subjected to four consecutive freeze-thaw cycles in chilled ethanol and 37 °C water bath for one minute each. Further, the protocol was same as described [31].

### Viability of vit C-treated THP-1 cells

Briefly, ~5 × 10^4^ THP-1 cells were seeded in a 96-well tissue culture plate in triplicate wells and differentiated with 30 nM PMA for 16-18 h. The cells were washed and allowed to rest for 2-3 h. This was followed by the addition of vit C (100 μM) and the viability of treated and control wells were assessed using MTT at 96 h. Briefly, 20 μL of MTT (Sigma-Aldrich^®^) (5 mg/mL) was added and incubated for 4–5 h at 37 °C. Following incubation, media was discarded and the formazan crystals were solubilized by adding 200 μL DMSO and the absorbance measured at 590 nm.

## Results

### Intracellular vit C accumulation

**Fig. 2:**
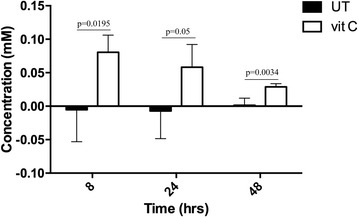
Intracellular concentration of vit C. PMA-differentiated THP-1 cells were treated with 100 μM vit C. The intracellular concentration of vit C was estimated in the control (untreated, UT) as well as vit C-treated cells. Mean ± SD is plotted from 3 biological replicates values. *P*-value was calculated using Two-tailed unpaired t-test (*p*-value less than or equal to 0.05 was considered to be significant)

Towards understanding the effect of physiological levels of vit C on macrophage gene expression, the intracellular accumulation of vit C was estimated in THP-1 cells. It was determined to be in the range of 20 to 80 μM, showing highest accumulation at 8 h followed by a decline till 48 h (Fig. 2). This low level is considered to be the baseline intracellular concentration of Vit C in THP-1 cells under these conditions. Vit C does not exert any toxic effect on THP-1 cell viability (Additional file 1: Figure S1).

### Temporal transcriptome analysis

**Fig. 3:**
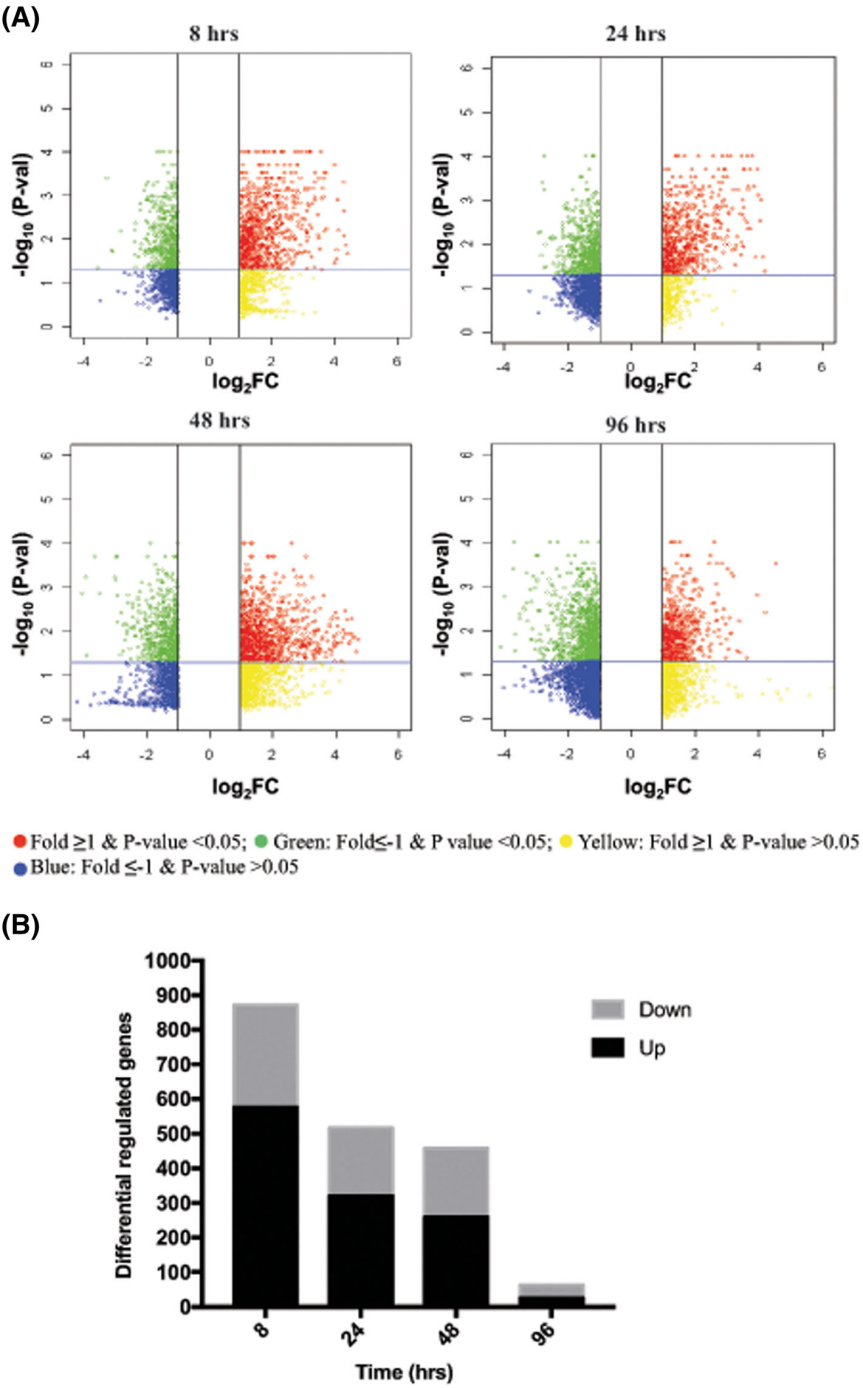
Differentially regulated genes (DRGs) on treatment with vit C. **a** Volcano plots depicting the magnitude and significance of differential expression at all time-points. Each dot on the plot is single gene. *Vertical lines* represent the fold change -1 ≤ 0 ≥ 1 and *horizontal lines* represent the *p*-value ≤0.05 **b** DRGs were determined using the absolute log_2_ fold change ≥1 (*p*-value ≤0.05, FDR ≤ 0.05)

Microarray RNA expression data was generated at 8, 24, 48 and 96 h from both vit C-treated and control PMA-differentiated THP-1 cells as described in Methods. Log_2_ transformed 75th percentile normalized data was analyzed by hierarchical clustering (Fig. 1b), establishing the robustness of the data. Further, the analysis of the expression of ten representative housekeeping genes and their raw gProcessed signal intensities (background subtracted) values showed the data to be normally distributed (Additional file 2: Figure S2a and S2b). Widespread changes in gene expression over time were noted upon treatment of differentiated THP-1 cells with physiological levels of vit C (Fig. 3a). The volcano plots reflect the extent of differential expression (absolute log_2_ fold change ≥1) and its significance; the red and green dots highlight the significantly differentially expressed genes (*p*-value ≤0.05) whereas the yellow and blue dots indicate the non-significant differential expression. Further, differentially regulated genes (DRGs) with absolute log_2_ fold change ≥1, *p*-value ≤0.05 with FDR correction ≤0.05, indicated a temporal reduction in the number of DRGs; at 8 and 24 h, there were 872 and 517 DRGs, respectively, and the number decreased to 63 at 96 h following vit C treatment (Fig. 3b). The expression of genes coding for GLUT and SVCT transporters were not induced in vit C-treated THP-1 cells; rather a modest down regulation at 8 and 96 h (nearly 2-fold repression) was noted. It was reasoned that the cells were exposed to physiological levels of vit C and moreover, they were not infected; therefore they are not required to accumulate vit C against the concentration gradient.

### Whole data enrichment analysis

Next, individual enrichment analysis was performed for the entire temporal data set. GOrilla enrichment tool was used for this analysis, employing the input option of a single ranked list of genes on the basis of expression values. The significantly enriched functional classes (*p*-value of enrichment ranging from 10^-4^ to 10^-15^ with FDR correction, q-value ranging from 10^-4^ to 10^-13^) were selected for gaining insights into global responses of THP-1 cells to vit C.

### Clustering of predominant functional responses

The functional enrichment is depicted as up- and down-regulated classes (described in sections below) to highlight the diversity in biological processes in response to vit C.

Further, the representation of the data does not exclude the possibility that different genes under a particular GO term respond in different directions. There are functional classes that showed significant enrichment under both up and down-regulated category thereby signifying the differential regulation of genes for the same biological process.

**Fig. 4:**
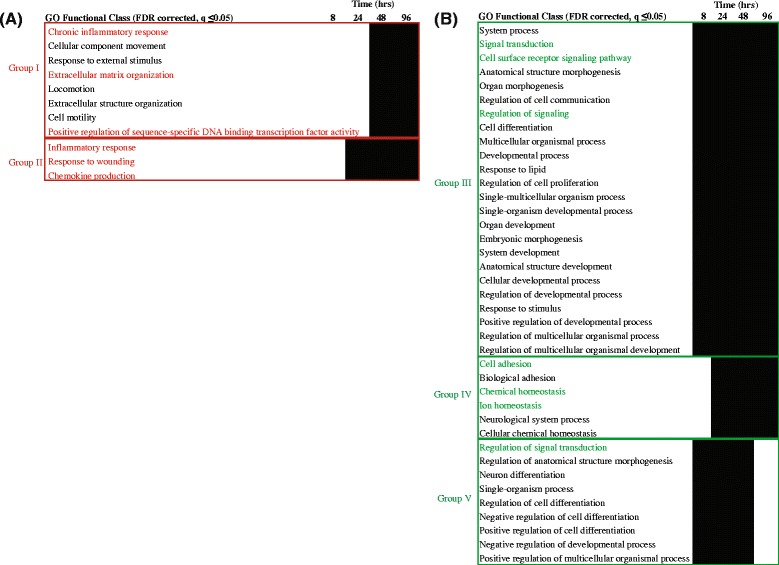
Clustering of enriched functional gene classes. Whole data enrichment analysis was performed using GOrilla, where the genes were ranked for up and down regulation. Enriched classes (filtered using *p*-value of enrichment <0.001, FDR corrected q ≤ 0.05) showing common temporal occurrence were grouped. **a** Up-regulated (*red*) GO classes classified into two groups, **b** Down-regulated (*green*) GO classes classified into three groups (*solid black boxes*)

The up-regulated functional classes were assigned into two groups (Fig. 4a): (1) up-regulated at late time-points *i.e.* 48 and 96 h (Group I: 8 functional classes), and (2), up-regulated from 24 h, onwards (Group II: 3 functional classes). Similarly, the down–regulated functional classes were categorized into three groups based on their temporal clustering pattern (Fig. 4b): (1) down-regulated at all the time-points (Group III: 24 functional classes), (2) common response at 24, 48 and 96 h (Group IV: 6 functional classes), and (3) common responses at 8, 24 and 48 h (Group V: 9 functional classes). In addition to these common responses, several functional classes were enriched uniquely at a specific time-point (discussed in a later section).

### Up-regulated cluster of functional classes

**Fig. 5:**
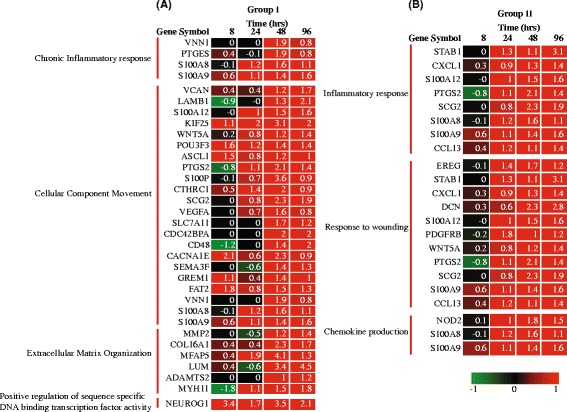
Gene expression profile of the up-regulated group of GO classes. Log_2_ fold expression values are shown of the genes falling under the mentioned GO classes that were commonly present (**a**) at 48 h and 96 h (Group I), (**b**) from 24 h onwards (Group II). Genes shown here are not filtered according to FDR corrected *p*-value, since these represent common occurrence. FDR corrected *p*-values for the genes ranged from 0.03 to 0.09

The functional classes in Groups I and II (Fig. 4a) are highly relevant in the context of macrophage function, especially in relation to infection. The GO class ‘Chronic inflammatory response’ reflected the role of vit C in immune function, and included 4 genes, VNN1, PTGES, S100A8 and S100A9, emphasizing their role in oxidative stress response, signaling pathways and in regulation of cytoskeleton (Fig. 5a). A second noteworthy up-regulated GO class is ‘Extracellular matrix (ECM) organization’ and included the genes MMP2, COL16A1, MFAP5, LUM, ADAMTS2 and MYH11, that play important roles in inflammation and in remodeling and maintaining the integrity of ECM (Fig. 5a). The third relevant class in Group I is ‘Positive regulation of sequence-specific DNA-binding transcription factor activity’, which included NEUROG1, that codes for a transcriptional regulatory protein (Fig. 5a). Further, the GO classes that showed sustained up regulation from 24 h onwards were classified into group II and included ‘Inflammatory response’ (8 genes), ‘Response to wounding’ (11 genes) and ‘Chemokine production’ (3 genes, Fig. 5b). The up regulation of these classes suggested a role for vit C in preparing macrophages for their role as the first line of defence.

### Down-regulated cluster of functional classes

**Fig. 6:**
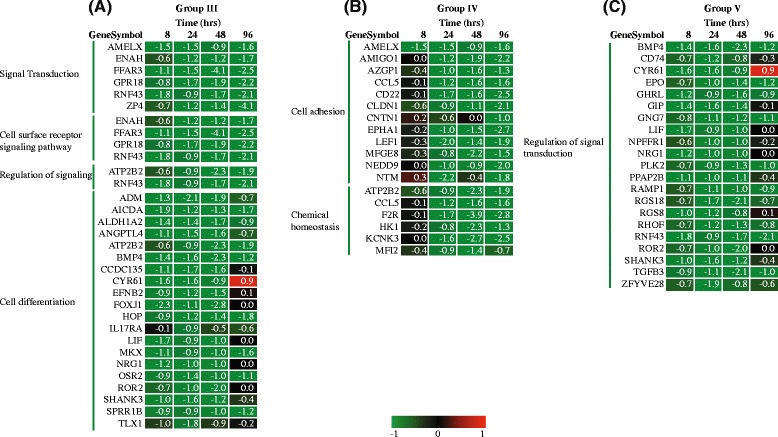
Gene expression profile of the down-regulated group of GO classes. Log_2_ fold expression values are shown of the genes falling under the mentioned GO classes that were (**a**) commonly present from 8 h to 96 h (Group III), (**b**) commonly present from 24 h onwards (Group IV), and (**c**) commonly present from 8 h to 48 h (Group V). Genes shown here are not filtered according to FDR corrected *p*-value, since these represent common occurrence. FDR corrected *p*-values for the genes ranged from 0.03 to 0.09

Similarly, the classes that were down-regulated in response to vit C were classified into 3 groups (Fig. 4b). Group III constitutes a large number of GO classes that were persistently down-regulated at all the four time-points. Many of these classes were from the top-level hierarchy (‘System process’, ‘Anatomical structure morphogenesis’ etc.) and therefore, highlight a more broad function and are not discussed in detail. The classes relevant to the scope of the present study are discussed below and included ‘Signal transduction’, ‘Cell surface receptor signaling pathway’ and ‘Regulation of signaling’, reflecting the significant extent of modulation at transcriptional level exerted by vit C. The genes among the classes included FFAR3, ZP4, ENAH, AMELX, RNF43 and GPR18 (Fig. 6a) involved in energy homeostasis, cytoskeleton remodeling and important in the regulation of immune system. The group IV cluster includes enriched functional classes down-regulated from 24 h onwards (Figs. 4b and 6b), namely, ‘Cell adhesion’, ‘Biological adhesion’, ‘Chemical homeostasis’, ‘Ion homeostasis’ and ‘Cellular chemical homeostasis’. These GO classes are structured at different levels of hierarchy and mainly fall into ‘Cell adhesion’ and ‘Chemical homeostasis’ at the top level of hierarchy. The down regulation of genes in ‘Cell adhesion’ class (such as AMIGO1, CCL5, CNTN1, NEDD9 and NTM genes) suggested the role of vit C in regulating signaling complexes important in cell attachment, migration and invasion as well as apoptosis and cell cycle. The class ‘Chemical homeostasis’ belongs to a higher level of hierarchy and covers ‘Ion homeostasis’ and ‘Cellular chemical homeostasis’ (Fig. 4b). Group V *i.e.* functional response common from 8 to 48 h, is mainly represented by the class ‘Regulation of signal transduction’ (Fig. 6c). As mentioned above and explained in later sections also, although the classes have an overlap, they clustered differently based on the temporal expression of discrete genes in each class. In summary, the common responses to vit C, both up and down-regulated, shed light on adaptation mechanisms employed by macrophages to prepare themselves for their defensive function. Vit C also seems to exert a regulatory role in maintaining homeostasis by regulating diverse activities such as inflammation, adhesion, signaling and ECM remodeling.

### Unique temporal responses

In addition to the common transcriptional response described above, several functional classes showed time-dependent expression following vit C treatment. The classes pertinent to the role of THP-1 cells in macrophage function are discussed in detail.

### Early response to vit C (at 8 h)

**Fig. 7:**
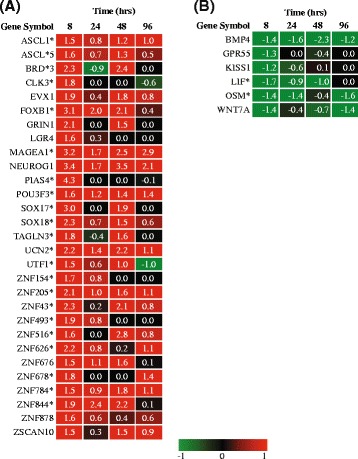
Gene expression profile of the enriched GO classes at 8 h post vit C treatment. **a** ‘Regulation of Gene expression’ **b** ‘Positive regulation of MAPK cascade’. The values of the genes represent the log_2_ fold change ≥1 (*p*-value ≤0.05), asterisk marked genes have FDR q ≤0.05. Up-regulated and down-regulated classes are indicated in *red* and *green*, respectively

One of the relevant classes enriched at the earliest time-point studied was ‘Regulation of gene expression’. This enriched class includes a large number of genes coding for zinc finger proteins that act as transcriptional regulators as well as other regulatory genes such as ASCL1 and POU3F3. Few other classes that were functionally linked to the ‘Regulation of gene expression’ were also enriched *i.e.* ‘Transcription DNA-dependent’, ‘mRNA transcription from RNA pol II promoter’ and ‘Regulation of RNA biosynthetic process’. All of these classes were analyzed for the temporal expression values of the respective genes and many of the genes were commonly present in these classes. The temporal expression behavior of these genes showed upregulation at 8 h (Log_2_ fold ≥1, *p*-value ≤0.05 and q ≤0.05) (Fig. 7a) and subsequent decrease in expression. This indicated that vit C induces early extensive gene expression changes at the transcriptional level, suggesting its role in preparing cells for a future event, such as an infection. The expression of target genes for some of the up-regulated transcriptional regulators (derived from the database HTRIdb, http://www.lbbc.ibb.unesp.br/htri) was lowered at later time points. For example: the down-regulated target genes of ASCL1 *i.e.* DKK1, IGF2 and PCSK6, are involved in ‘Regulation of gene expression’ and ‘Regulation of Signal transduction’ thereby suggesting the early effect of vit C on transcription, hence modulating the expression of many target genes falling into diverse biological processes at later time points till 96 h. A brief analysis of the induced transcriptional regulators and their target gene expression is shown in Additional file 3: Table S1.

The enrichment result for the down-regulated classes revealed that the majority of the classes were not obviously linked to the function of macrophages under ex vivo condition. An exceptional class *‘*Positive regulation of MAPK cascade’ (Fig 7b) is discussed, as it is under ‘Regulation of signal transduction’ class, which seemed to be a continuous response to vit C (Fig. 4b). The function of these genes (Fig. 7b) covers a wide spectrum of functional processes such as growth and differentiation, cell survival and macrophage function, some genes play an essential role in innate immune response and inflammation and are involved in cytoskeleton reorganization.

### Response at 24 h

Three major biological processes predominate the functional response to vit C at 24 h. The enriched GO classes broadly fall into ‘Cell cycle’, ‘Chromosome organization’ and ‘DNA metabolic process’ at a higher level of hierarchy (Table 1). Out of 87 GO classes enriched at 24 h, 17 classes (*p* ≤ 0.001 and q ≤0.05) belong to ‘Cell cycle’, 11 classes (*p* ≤ 0.001 and q ≤0.05) belong to ‘Chromosome organization’ and 18 classes (*p* ≤ 0.001 and q ≤0.05) belong to ‘DNA metabolic process’ (Table 1). Using the 2-fold criterion and corrected *p*-value ≤0.05, the class ‘Cell cycle check point’ contained genes, namely, CDC7, DTL, BLM and MCM10. Similarly, another class in Cell cycle category is ‘Cell cycle phase transition’ and included genes CDC7, MCM10. Some of the genes directly play a role at the mitotic spindle (LRRCC1), others regulate at the level of DNA replication (CDC7, BLM, MCM10). The enrichment of as many as 17 GO classes belonging to the ‘Cell cycle’ and ‘Biological phase’ at higher hierarchy indicate the significant regulatory role of vit C in cell cycle. The second category ‘Chromosome organization’ included 11 GO classes (Table 1). The first in the list, ‘ATP dependent chromatin remodeling’, contains genes *i.e.* MLF1IP and CENPK. These genes play an important role in centromere assembly (MLF1IP) along with CENPK. The third major representative class was ‘DNA metabolic process’, which included a total of 18 classes, such as ‘DNA repair’, ‘DNA recombination’ and ‘DNA replication’ (Table 1). In conclusion, the response of THP-1 cells at 24 h was predominantly linked to cell cycle regulation via regulating events at phases of cell cycle, chromosome remodeling and DNA metabolism. Importantly, the functional cellular responses associated with signaling appear to be under the regulation of vit C. The classes ‘Positive regulation of signal transduction’ and ‘Negative regulation of signal transduction’ both showed enrichment; in fact this regulatory role of vit C was observed from 8 h onwards.

**Table 1:** Predominant enriched GO classes at 24 h in vit C-treated THP-1 cells

GO Term	Description	Higher hierarchy^a^	*P*-value*	FDR q-value^b^
GO:0007049	Cell cycle	Cell cycle	3.41E-09	0.000
GO:0000075	Cell cycle checkpoint	Cell cycle	1.76E-06	0.001
GO:0022403	Cell cycle phase	Biological phase	8.15E-13	0.000
GO:0044770	Cell cycle phase transition	Cell cycle	2.75E-06	0.001
GO:0022402	Cell cycle process	Cell cycle	6.52E-15	0.000
GO:0031570	DNA integrity checkpoint	Cell cycle	1.33E-05	0.004
GO:0000082	G1/S transition of mitotic cell cycle	Cell cycle	8.40E-05	0.019
GO:0000279	M phase	Biological phase	2.25E-04	0.043
GO:0000087	M phase of mitotic cell cycle	Cell cycle	1.89E-04	0.039
GO:0007126	Meiosis	Cell cycle	3.44E-06	0.001
GO:0007067	Mitosis	Cell cycle	2.06E-04	0.041
GO:0000278	Mitotic cell cycle	Cell cycle	7.08E-13	0.000
GO:0044772	Mitotic cell cycle phase transition	Cell cycle	2.75E-06	0.001
GO:0000083	Regulation of transcription involved in G1/S phase of mitotic cell cycle	Cell cycle	1.34E-06	0.001
GO:0051320	S phase	Biological phase	6.16E-06	0.002
GO:0000084	S phase of mitotic cell cycle	Cell cycle	1.62E-05	0.005
GO:0032201	Telomere maintenance via semi-conservative replication	Cell cycle	5.83E-07	0.000
GO:0043044	ATP-dependent chromatin remodeling	Chromosome organization	5.29E-06	0.002
GO:0034080	CENP-A containing nucleosome assembly at centromere	Chromosome organization	3.34E-07	0.000
GO:0006333	Chromatin assembly or disassembly	Chromosome organization	7.41E-06	0.003
GO:0031055	Chromatin remodeling at centromere	Chromosome organization	4.74E-07	0.000
GO:0006336	DNA replication-independent nucleosome assembly	Chromosome organization	3.34E-07	0.000
GO:0034724	DNA replication-independent nucleosome organization	Chromosome organization	3.34E-07	0.000
GO:0043486	Histone exchange	Chromosome organization	3.12E-06	0.001
GO:0006334	Nucleosome assembly	Chromosome organization	2.18E-06	0.001
GO:0034728	Nucleosome organization	Chromosome organization	3.98E-05	0.011
GO:0010833	Telomere maintenance via telomere lengthening	Chromosome organization	5.60E-05	0.014
GO:0032200	Telomere organization	Chromosome organization	5.83E-05	0.014
GO:0006259	DNA metabolic process	DNA metabolic process	2.44E-10	0.000
GO:0006310	DNA recombination	DNA metabolic process	6.21E-07	0.000
GO:0006281	DNA repair	DNA metabolic process	1.15E-08	0.000
GO:0006260	DNA replication	DNA metabolic process	4.57E-14	0.000
GO:0006270	DNA replication initiation	DNA metabolic process	5.44E-07	0.000
GO:0022616	DNA strand elongation	DNA metabolic process	1.47E-14	0.000
GO:0006271	DNA strand elongation involved in DNA replication	DNA metabolic process	3.82E-15	0.000
GO:0006268	DNA unwinding involved in replication	DNA metabolic process	2.23E-04	0.044
GO:0006261	DNA-dependent DNA replication	DNA metabolic process	2.36E-05	0.007
GO:0000724	Double-strand break repair via homologous recombination	DNA metabolic process	8.53E-06	0.003
GO:0045005	Maintenance of fidelity involved in DNA-dependent DNA replication	DNA metabolic process	9.31E-05	0.020
GO:0006312	Mitotic recombination	DNA metabolic process	7.17E-06	0.003
GO:0006297	Nucleotide-excision repair, DNA gap filling	DNA metabolic process	3.51E-05	0.010
GO:0000725	Recombinational repair	DNA metabolic process	1.28E-05	0.004
GO:0051052	Regulation of DNA metabolic process	DNA metabolic process	1.02E-05	0.003
GO:0006275	Regulation of DNA replication	DNA metabolic process	7.56E-05	0.018
GO:0000723	Telomere maintenance	DNA metabolic process	4.14E-05	0.011
GO:0000722	Telomere maintenance via recombination	DNA metabolic process	2.71E-06	0.001

*p-value, significance value of Gene ontology enrichment (determined in GOrilla analysis)

^a^Each enriched GO term at 24 h is listed with the higher hierarchy GO term as determined from the Amigo 2 tree view (amigo.geneontology.org/amigo)

^b^FDR q-value, *p*-values after multiple testing correction

### Responses at 48 h

**Fig. 8:**
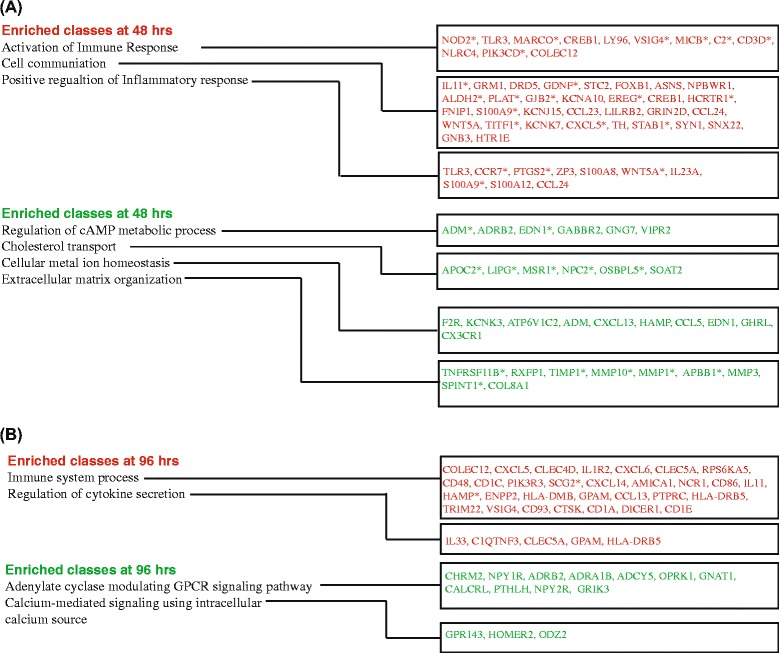
Sustained functional responses of THP-1 to vit C-treatment. Functional classes enriched at (**a**) 48 h and (**b**) 96 h are shown. The genes mentioned for each class, induced (*red*) or repressed (*green*), were obtained using log_2_ fold change ≥1 (*p*-value ≤0.05), genes marked with asterisk have FDR q ≤0.05

Proceeding to later time-points, the identity of the most represented classes provided clues to the immune-modulatory role of vit C. The prominent functional classes included ‘Activation of immune response’, ‘Positive regulation of immune response’, ‘Positive regulation of inflammatory response’ and ‘Cell communication’. There is an extensive gene overlap among the classes ‘Positive regulation of immune response’ and ‘Activation of immune response’, therefore; only one class is mentioned (Fig. 8a). The enrichment of these classes suggests that vit C likely plays an indispensable role in macrophages function in innate defence *e.g.* up regulation of genes coding for innate receptors (NOD2, TLR3, MARCO, COLEC12), and associated proteins that co-operate with TLR in response to bacterial LPS (LY96). In the same way, the genes involved in immune response *i.e.* part of inflammasome (NLRC4) and signaling protein (PIK3CD) indicated the fundamental role of vit C in the immune system. Interestingly, CREB1 was up-regulated at 48 h; CREB has been shown to induce the transcription of immune-related genes that possess a CRE element and hence, one of the ways in which vit C exerts its immune modulatory effect is via CREB. The enriched classes that showed down regulation at 48 h were ‘Regulation of cAMP metabolic process’, ‘Cholesterol transport’, ‘Cellular metal ion homeostasis’ and ‘Extracellular matrix organization’ (Fig. 8a). In view of the earlier described enriched class ‘Regulation of gene expression’, the enrichment of the GO class ‘Regulation of cAMP metabolic process’ was considered relevant. The down-regulated genes included those associated with the activation of adenylate cyclases (*e.g.* ADRB2, GNG7, VIPR2) and with the inhibition of adenylate cyclase, GABBR2 (Fig. 8a). This is suggestive of the regulatory effect of vit C on adenylate cyclase activity, thereby possibly modulating the levels of cAMP. The down regulation of genes under the class ‘Cholesterol transport’ (APOC2, LIPG, MSR1, NPC2, OSBPL5, SOAT2) suggested the modulation in the uptake (MSR1), lipase activity (LIPG) and egress (NPC2) of cholesterol on vit C treatment. This emphasized the importance of vit C in maintaining cholesterol balance. One interesting finding was the enrichment of class ‘Extracellular matrix organization’. The role of vit C in ECM remodeling has already been discussed under common up-regulated group of genes (Fig. 5a), and here the class was enriched under down regulation. This suggested that different choices are exerted in terms of genes regulation, depending upon the requirement of the cell *e.g.* at 48 h COL16A1 was up-regulated and COL8A1 was down-regulated (Fig. 8a). Also, at 48 h the selection of MMPs were different (Fig. 5a and Fig. 8a). The enrichment of the class ‘Cellular metal ion homeostasis’ emphasizes the important role of vit C in maintaining ion equilibrium inside the macrophages. This class included genes coding for receptors (F2R) and ion channels (KCNK3). In addition, the gene involved in the maintenance of iron homeostasis and for the regulation of iron storage in macrophages, was also down-regulated on vit C treatment (HAMP, Fig. 8a). As seen from the analysis, vit C treatment affected functional responses related to the maintenance of homeostasis, which is not surprising. Rather, the novelty lies in the gamut of regulatory roles played by vit C, at least at the transcriptional level.

### Responses at 96 h

Persisting from 48 h onwards were the ‘Immune system process’ and ‘Regulation of cytokine secretion’ classes that reflect vit C to be of paramount importance for immune system function (Fig. 8b). Consistent with this role, the up-regulated classes at 96 h include genes coding for chemokines, coding for molecules involved in antigen presentation (Fig. 8b). Thus, vit C exerts its regulatory role on immune recognition (CD1 and HLA) as well as on levels of cytokines/chemokines, thereby aiding macrophages’ role in innate defence. Importantly, the expression pattern at the later time-point of the study *i.e.* 96 h, is indicative of a sustained cellular response to vit C.

Among the down-regulated classes, the enrichment of the class ‘Adenylate cyclase modulating GPCR signaling pathway’ indicated a sustained modulatory effect of vit C on cAMP levels and associated signaling (Fig. 8b). A careful examination of the genes involved, revealed the down regulation of genes responsible for the activation of adenylate cyclase (*e.g.* ADRB2, CALCRL) and also, the inhibition of adenylate cyclase activity (*e.g.* CHRM2, OPRK1, NPY1R). The plausible underlying explanation could be that it is the balance in the activities of these opposing functions, depending on the stimuli and their duration, which ultimately determines the intracellular levels of cAMP. Linked to the class ‘Chemical ion homeostasis’ described at 48 h, the down regulation of ‘Calcium related signaling using intracellular calcium source’ indicated the modulation of calcium ion-associated signaling pathways on vit C-treatment. The decreased expression of HOMER2 (interacts with Ryanodine receptors (RYR)) and also of the GPCR *i.e.* GPR143 that stimulates Ca^2+^ influx into the cytoplasm suggested modulatory effect of vit C on calcium-associated signaling (Fig. 8b).

It is imperative to emphasize that although the temporal response to vit C was analyzed in a step-wise manner, the functional responses clearly reflected an interconnection of the biological pathways, that are integrated in a whole biological system.

## Discussion

The biological role of dietary antioxidant molecules is no longer simply ascribed to their ability to serve as ‘electron donors’; rather, antioxidants such as vit C in the present context, also act by modulating gene expression and signaling. In vivo and in vitro studies have shown that some of the effects of vit C on cells are at the transcriptional level [32–34]. The present study is the first report of the genome-wide effects of vitamin C on gene expression of differentiated THP-1 cells treated with a physiologically relevant concentration of vit C, namely circulating plasma concentration of *~* 100 μM. The intracellular concentration of vit C in THP-1 cells ranged between 20 μM and 80 μM during 8 to 48 h. Macrophages are expected to be constantly exposed to ~ 70 to 100 μM levels of vit C in nutritionally adequate subjects [5], therefore, the observed modulation of gene expression can be considered as a baseline macrophage-like cell response to physiological concentrations of vit C. It is remarkable that this baseline concentration of vit C mediates widespread changes in gene expression of THP-1 cells as revealed by whole genome enrichment analysis.

**Fig. 9:**
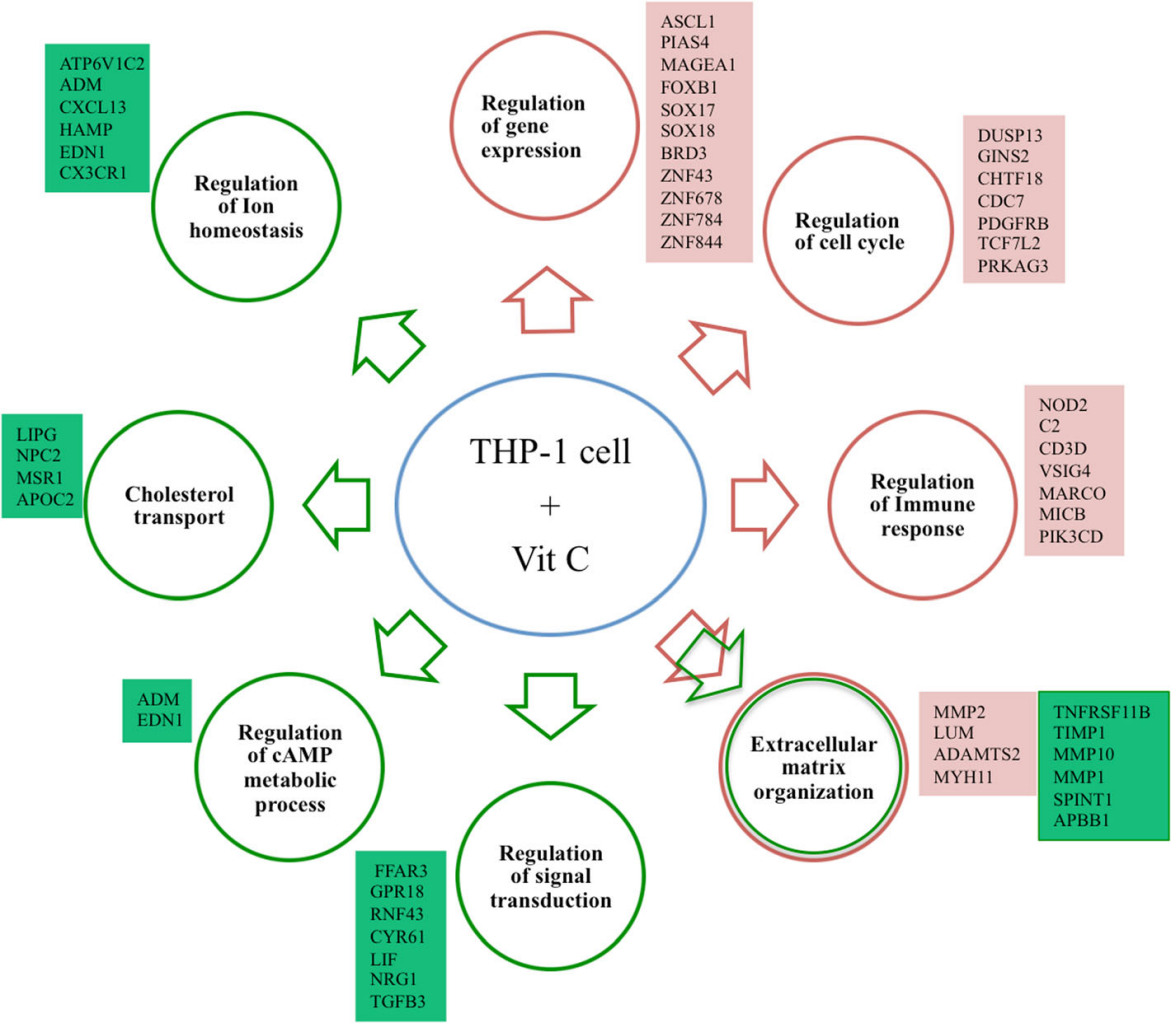
Vit C regulates a wide spectrum of biological processes. The enriched functional processes that are up-regulated (*red*) and down-regulated (*green*) along with the top induced or repressed genes (log_2_ fold change ≥1, with FDR corrected *p*-value ≤0.05) in the same color code are shown. *Double colored arrow* indicates the enriched class under both up- and down-regulation

The major physiological processes modulated on vit C treatment in THP-1 cells are represented in Fig. 9. The earliest response (8 h) to vit C was reflected in the enriched ‘Regulation of gene expression’ GO class. The majority of the genes included in this class are transcriptional regulators. The target genes of some of these regulators (Fig. 7, Additional file 3: Table S1) function in diverse processes such as ECM organization and signal transduction. This was consistent with the recognized role of vit C in increasing collagen gene transcription in various cell types such as chondrocytes and skin fibroblasts, [35–39], stabilizing collagen mRNAs [40] and increasing procollagen secretion [41]. This suggests that ascorbate action not only involves hydroxylation and stabilization of the collagen triple helix [42], it also involves direct or indirect effects on gene expression and protein secretion. ‘Regulation of cAMP metabolic process’ was detected as an enriched class from 48 h onwards, which pointed towards the modulatory effects of vit C on cAMP, consistent with vit C being proposed as a ‘global regulator’ of intracellular cAMP [43, 44]. This advances the possibility that the modulatory effects of vit C on cAMP might be one of the mechanisms by which vit C regulates gene expression.

Vit C seemed to exert its effect on other notable functional processes in THP-1 cells as well, namely, ‘Cell cycle’, ‘DNA replication’ and ‘Chromosomal organization’ (Table 1) at 24 h and included genes belonging to various GO classes reflecting role of vit C at different steps in the cell cycle such as cell cycle check point, G1 to S transition, genes involved in DNA replication and repair as well as genes involved in chromosomal remodeling. Many reports have explained the effect of vit C on cell cycle processes [45, 46]. Similarly, Belin et al. demonstrated the anti-proliferative role of vit C, potentially due to the inhibition of expression of genes involved in cell division progression [47]. Another noteworthy response to vit C was ‘Extracellular matrix organization’ biological process. ECM organization is a well-known process where vit C plays an essential role. This class was enriched in both up and down regulation (summarized in Fig. 9) and is consistent with the essential cofactor function of vit C in the synthesis of collagen, the main structural component of ECM. Vit C is required for the hydroxylation of proline residues in collagen chains by prolyl hydroxylase [48], for proper triple helix assembly in the endoplasmic reticulum and for the secretion of procollagen [49]. A study of skin fibroblasts reported the modulatory effects of a stable vit C derivative (AA2P) on ECM remodeling [50]. A comparison of the gene profiles of THP-1 and skin fibroblast cells revealed a similar GO enrichment analysis with a differential selection of genes in these two cell types for the same physiological response. For example, IL-6 is down-regulated in THP-1 cells, whereas this gene is induced in skin fibroblasts [50].

‘Modulation of Immune response’ is one of the most noteworthy functions of vit C (48 h onwards) [51–55]. Vit C appears to mediate the regulation of various aspects of the immune response, namely, innate receptors, chemokines, antigen presentation, immune signaling and transcriptional regulation. Another interesting class where vit C exerted its effect was ‘Cholesterol transport’. The association between cholesterol metabolism and vit C was observed when chronic latent vitamin C deficiency led to hypercholesterolaemia and cholesterol accumulation in certain tissues [56], suggesting that deficiency of vitamin C might deregulate cholesterol homeostasis. The THP-1 cell infection model is widely considered as a suitable model for studies of Mtb infection [21, 23]. Interestingly, Mtb infection results in the acquisition of a foam cell phenotype [57], wherein, Mtb maintains itself within lipid bodies and develops drug tolerance [57, 58]. THP-1 cells have also been established as a model to study atherosclerosis due to its foam cell phenotype [59]. Enrichment analysis in the present study showed down regulation of genes (Fig. 8a) participating in lipase activity (ApoC2, LIPG), uptake (MSR1) and egress (NPC2). Further analysis of ATP-binding cassette transporters, ABCA1 and ABCG1, which play a pivotal role in cholesterol efflux from macrophage foam cells [60], revealed no significant change in expression. Additionally, nuclear receptors, peroxisome proliferator activated receptors (PPARs) known to exert anti-atherogenic effects by enhancing cholesterol efflux via activation of the liver X receptor (LXR)-ABCA1 pathway [61, 62], showed up regulation by nearly 2-fold at 48 h (PPARα and PPARγ). At physiological levels, the function of vit C in THP-1 cells appears to be more towards balancing the uptake and efflux of cholesterol and thereby preventing the formation of foam cells.

A novel role of vit C was observed to be in ‘Cellular metal ion homeostasis’, particularly in calcium homeostasis as evident by the enrichment of the class ‘Calcium-mediated signaling using intracellular calcium source’ (at 96 h). The decreased expression of genes involved in calcium associated signaling such as GPR143 and HOMER2, indicated the regulation of calcium ion on vit C treatment. There is no report in the literature to the best of our knowledge that has described this regulatory role of vit C. Another ion of interest is iron. Vit C within mammalian systems can regulate cellular iron uptake and metabolism, both at the transcriptional and post-transcriptional levels. The major regulatory factor for the transcriptional control of certain genes involved in iron metabolism is the HIF system that responds to changes in oxygen (O_2_) tension, intracellular iron and vit C levels. The HIF system includes the O_2_and iron-regulated proteins, HIF1α and HIF2α [63, 64]. Under conditions of normoxia, high levels of vit C (>100 μM) and iron, prolylhydroxylases are fully active and hydroxylate the 1α subunit at specific proline residues [65, 66], targeting HIF1α for proteasomal degradation [67, 68]. Under the condition of low O_2_ (3–5% O_2,_ in vivo), low iron, and low vit C, HIF1α /2α proteins are stabilized and activate the transcription of specific genes that contain hypoxia- response elements (HREs), such as genes encoding Tf(TF), TfR1 (TFRC). Interestingly, none of the HIF target genes (described by Lane and Richardson [69]) showed any change of expression in THP-1 cells on vit C-treatment, possibly owing to the differences in regulatory mechanism under cell culture and in vivo conditions [68, 70, 71].

A comparison with the responses of skin fibroblasts to [50] suggested that macrophages were far more responsive to treatment with vit C; nearly 294 genes were shown to be differentially regulated in skin fibroblasts at 5 days [50], in contrast, 874 genes were differentially regulated at the earliest time-point *i.e.* 8 h in THP-1 cells. The number of DRGs, however, decline from 8 to 96 h.

The absence of cellular response to oxidative stress was a notable finding of this study. It has been reported that pharmacological levels of vit C (0.3 mM to 20 mM) mediate Fenton chemistry that occurs readily in vitro and generates reactive oxygen species [72]. However, due to the non-availability of catalytic metal ions in vivo and in cell culture owing to their sequestration by various metal binding proteins such as ferritin, transferrin, and ceruloplasmin [73, 74], there are minimal chances of the occurrence of Fenton reaction [74]. Thus, the pro-oxidant effect of vit C can be ruled out in the present study as vit C was used at physiological levels and not at pharmacological levels [72, 75].

## Conclusions

In conclusion, this genome-wide transcriptome analysis has disclosed that vit C, being an essential dietary component, regulates a wide spectrum of biological processes in THP-1 macrophages. These insights have opened a new dimension to be explored towards understanding the pleiotropic effects of vit C on eukaryotic gene expression and function. The study will also have an impact on curating databases and biological networks. The present findings further point towards the potential utility of the THP-1 cell model for examining the role of vit C in modulating macrophage responses to various stresses, including infection by intracellular pathogens.

## Additional files

Additional file 1: Figure S1. Viability of vit C –treated THP-1 cells at 96 h. PMA-differentiated THP-1 cells were treated with 100 μM vit C in 96-well plate format. At 96 h post vit C-treatment, viability was assessed using MTT assay and A_590_ was measured. UT represents untreated control. Mean ± SD is plotted from six readings. The difference was not significant (*p* = 0.6) as calculated using Two-tailed unpaired t-test. (DOCX 93 kb)

Additional file 2: Figure S2a. Fold expression values for the housekeeping genes. Heat map was generated using the fold expression values for ten housekeeping genes. **Figure S2b.** Raw gProcessed signal intensity values for housekeeping genes. The values shown on the Y-axis (log_2_ scale) are the background subtracted raw intensity values. UT, untreated; AA, ascorbic acid (vit C- treatment). The numbers in sample names refer to biological replicate numbers. (DOCX 262 kb)

Additional file 3: Table S1. Target gene expression of regulatory genes enriched in class ‘Regulation of gene expression’ at 8 h. (DOCX 95 kb)
