# Supplementary material for: Genome-wide expression profiling establishes novel modulatory roles of vitamin C in THP-1 human monocytic cell line
Source: BMC Genomics. 2017 Mar 23;18:252. doi: 10.1186/s12864-017-3635-4 (PMC5364625; doi:10.1186/s12864-017-3635-4)
Supplement: Supplementary file 1 — Viability of vit C –treated THP-1 cells at 96 h. PMA-differentiated THP-1 cells were treated with 100 μM vit C in 96-well plate format. At 96 h post vit C-treatment, viability was assessed using MTT assay and A590 was measured. UT represents untreated control. Mean ± SD is plotted from six readings. The difference was not significant (p = 0.6) as calculated using Two-tailed unpaired t-test. (DOCX 93 kb) [file 12864_2017_3635_MOESM1_ESM.docx]

**Additional File 1**

**Genome-wide expression profiling establishes novel modulatory roles of vitamin C in THP-1 human monocytic cell line.**

**Figure S1**

**Figure S1. Viability of vit C –treated THP-1 cells at 96 hrs.** PMA-differentiated THP-1 cells were treated with 100 μM vit C in 96-well plate format. At 96 hrs post vit C-treatment, viability was assessed using MTT assay and A_590_ was measured. UT represents untreated control. Mean±SD is plotted from six readings. The difference between UT and vit C was not significant (p=0.6) as calculated using Two-tailed unpaired t-test.
