# Supplementary material for: Genome-wide expression profiling establishes novel modulatory roles of vitamin C in THP-1 human monocytic cell line
Source: BMC Genomics. 2017 Mar 23;18:252. doi: 10.1186/s12864-017-3635-4 (PMC5364625; doi:10.1186/s12864-017-3635-4)
Supplement: Supplementary file 2 — Fold expression values for the housekeeping genes. Heat map was generated using the fold expression values for ten housekeeping genes. Figure S2b. Raw gProcessed signal intensity values for housekeeping genes. The values shown on the Y-axis (log2 scale) are the background subtracted raw intensity values. UT, untreated; AA, ascorbic acid (vit C- treatment). The numbers in sample names refer to biological replicate numbers. (DOCX 262 kb) [file 12864_2017_3635_MOESM2_ESM.docx]

**Additional File 2**

**Genome-wide expression profiling establishes novel modulatory roles of vitamin C in THP-1 human monocytic cell line.**

**Figure S2a**


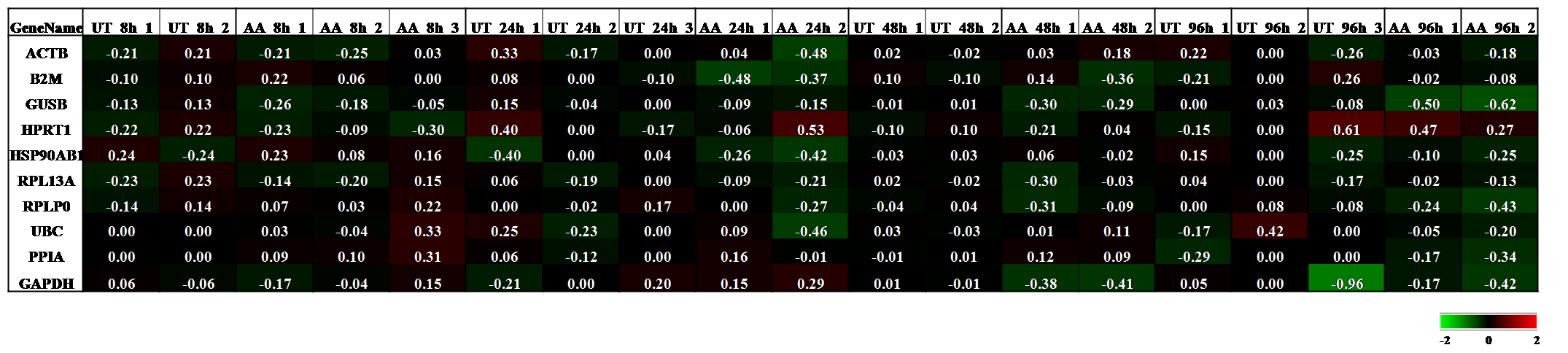


**Figure S2a. Fold expression values for ten housekeeping genes.** Heat map was generated using the fold expression values for ten housekeeping genes.

UT, untreated; AA, ascorbic acid (vit C- treatment). The numbers in sample names refer to biological replicate numbers.

**Figure S2b**

**
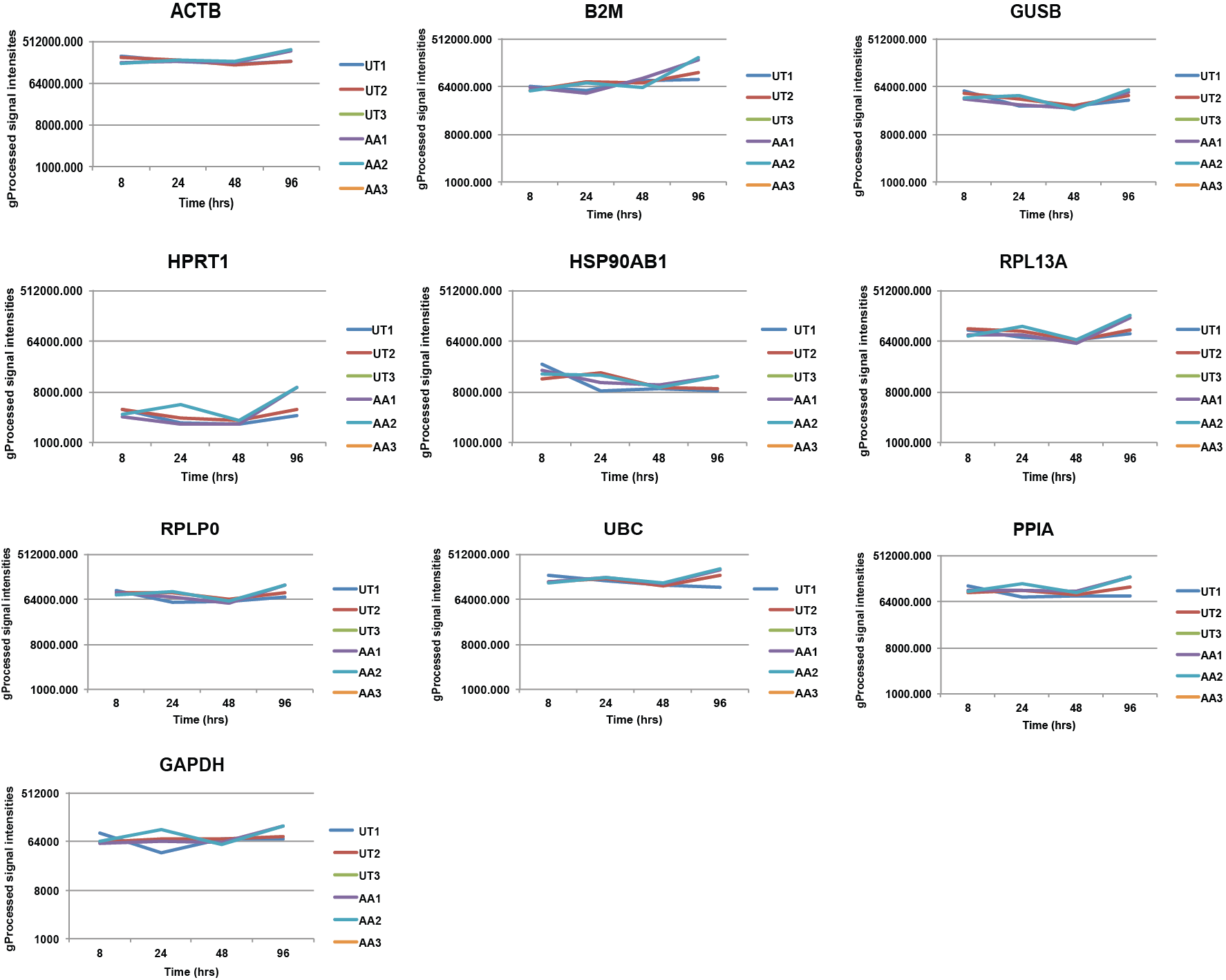
**

**Figure S2b. Raw gProcessed signal intensity values for ten housekeeping genes.** The values shown on the Y-axis (log_2_ scale) are the background subtracted raw intensity values. UT, untreated; AA, ascorbic acid (vit C- treatment). The numbers in sample names refer to biological replicate numbers.
