# Supplementary material for: Genome-wide expression profiling establishes novel modulatory roles of vitamin C in THP-1 human monocytic cell line
Source: BMC Genomics. 2017 Mar 23;18:252. doi: 10.1186/s12864-017-3635-4 (PMC5364625; doi:10.1186/s12864-017-3635-4)
Supplement: Supplementary file 3 — Target gene expression of regulatory genes enriched in class ‘Regulation of gene expression’ at 8 h. (DOCX 95 kb) [file 12864_2017_3635_MOESM3_ESM.docx]

**Additional File 3**

**Genome-wide expression profiling establishes novel modulatory roles of vitamin C in THP-1 human monocytic cell line.**

**Table S1: Target gene expression of regulatory genes enriched in class ‘Regulation of gene expression’ at 8 hrs**

|  |  |  | **Target Gene expression, Time (hrs)** | | | |  |
| --- | --- | --- | --- | --- | --- | --- | --- |
| **Regulator*** | **Target^#^** | **GO term (Biological processes)^$^** | **8** | **24** | **48** | **96** | **Reference** |
| ASCL1 | DKK1 | Regulation of gene expression | 0.3 | -0.7 | 0.1 | -1.0 | [1] |
| ASCL1 | IGF2 | Positive regulation of MAPK cascade, Regulation of gene expression | 0.0 | -0.7 | 0.0 | -1.0 | [2] |
| ASCL1 | PCSK6 | Regulation of gene expression, Regulation of signal transduction | -0.4 | -2.0 | -0.6 | -1.6 | [3] |
| ASCL1 | TPH1 | Oxidation reduction process | 0.2 | -0.6 | 0.2 | -1.3 | [1] |
| EVX1 | GSC | Regulation of gene expression | 0.0 | 0.0 | -0.5 | -1.5 | [4] |
| PIAS4 | MUC1 | Cellular homeostasis | -0.1 | 0.5 | -0.2 | -0.6 | [5] |
| PIAS4 | VDR | Regulation of gene expression, Cellular calcium ion homeostasis | -0.6 | -0.2 | -0.6 | -0.6 | [6] |
| SOX17 | HNF4A | Regulation of gene expression, Lipid metabolic process | 0.0 | -0.6 | 0.0 | -1.0 | [7] |
| SOX18 | MMP7 | Extracellular matrix organization | 0.3 | 1.4 | 0.2 | -1.2 | [8] |

*indicates the genes (log_2_ fold ≥1, p-value ≤0.05) enriched in the class Regulation of gene expression at 8 hrs **(Fig. 7)**

**^#^**Target indicates the genes identified from the literature to be under regulatory control of respective regulators ^$^GO term indicates the gene ontology term under the Biological process hierarchy. The values indicate the log_2_ fold expression of target genes.

**References:**

1. Johansson TA, Westin G, Skogseid B: **Identification of Achaete-scute complex-like 1 (ASCL1) target genes and evaluation of DKK1 and TPH1 expression in pancreatic endocrine tumours**. *BMC Cancer* 2009, **9**:321.

2. Li J, Neumann I, Volkmer I, Staege MS: **Down-regulation of achaete-scute complex homolog 1 (ASCL1) in neuroblastoma cells induces up-regulation of insulin-like growth factor 2 (IGF2)**. *Mol Biol Rep* 2011, **38**(3):1515-1521.

3. Yoshida I, Koide S, Hasegawa SI, Nakagawara A, Tsuji A, Matsuda Y: **Proprotein convertase PACE4 is down-regulated by the basic helix-loop-helix transcription factor hASH-1 and MASH-1**. *Biochem J* 2001, **360**(Pt 3):683-689.

4. Kalisz M, Winzi M, Bisgaard HC, Serup P: **EVEN-SKIPPED HOMEOBOX 1 controls human ES cell differentiation by directly repressing GOOSECOID expression**. *Dev Biol* 2012, **362**(1):94-103.

5. Brayman MJ, Dharmaraj N, Lagow E, Carson DD: **MUC1 expression is repressed by protein inhibitor of activated signal transducer and activator of transcription-y**. *Mol Endocrinol* 2007, **21**(11):2725-2737.

6. Jena S, Lee WP, Doherty D, Thompson PD: **PIAS4 represses vitamin D receptor-mediated signaling and acts as an E3-SUMO ligase towards vitamin D receptor**. *J Steroid Biochem Mol Biol* 2012, **132**(1-2):24-31.

7. Takayama K, Inamura M, Kawabata K, Katayama K, Higuchi M, Tashiro K, Nonaka A, Sakurai F, Hayakawa T, Furue MK *et al*: **Efficient generation of functional hepatocytes from human embryonic stem cells and induced pluripotent stem cells by HNF4alpha transduction**. *Mol Ther* 2012, **20**(1):127-137.

8. Hoeth M, Niederleithner H, Hofer-Warbinek R, Bilban M, Mayer H, Resch U, Lemberger C, Wagner O, Hofer E, Petzelbauer P *et al*: **The transcription factor SOX18 regulates the expression of matrix metalloproteinase 7 and guidance molecules in human endothelial cells**. *PLoS One* 2012, **7**(1):e30982.
